# One-year outcome after open inguinal hernia repair with self-fixated mesh: a randomized controlled trial

**DOI:** 10.1007/s00423-023-03106-w

**Published:** 2023-09-21

**Authors:** Anna-Maria Thölix, Jyrki Kössi, Jukka Harju

**Affiliations:** 1 Department of Abdominal Surgery, University of Helsinki and Helsinki University Hospital, Helsinki, Finland; 2 Department of Surgery, Päijät-Häme Central Hospital, Lahti, Finland

**Keywords:** Inguinal hernia, Open surgery, Mesh fixation, Chronic pain

## Abstract

**Purpose:**

The aim of this study was to analyze pain after surgery with the use of self-fixated meshes, which are commonly used as an alternative for sutured mesh in open inguinal hernia repair.

**Methods:**

This prospective randomized clinical trial was conducted from November 2018 to March 2021, with a follow-up duration of 12 months. Male patients, aged 18–85, and suitable for day case surgery, were included. The patients received the self-adhesive Adhesix™ mesh or the self-gripping Progrip™ mesh in open inguinal hernia surgery. The primary outcome was the number of additional follow-up visits due to post-operative pain. Secondary outcomes included intensity of pain, quality of life measures, and complications.

**Results:**

Two hundred seventy patients were included in this trial, 132 with Adhesix™ (A group), and 138 with Progrip™ (P-group). All patients’ medical records were reviewed 12 months after surgery, and 207 patients (76.2%) completed 12-month follow-up. The number of patients needing additional follow-up visits 3–12 months after surgery were comparable (A group 3/3.0%, P-group 6/5.6%). The numeric rating scale was low at 12 months after surgery (at rest A 0.21, P 0.34, at exercise A 0.78, P 0.90). The incidence of chronic pain, that is moderate or severe pain during exercise, was 5 patients (5.2%) with Adhesix™ and 8 patients (7.4%) with Progrip™ (*P* = 0.333). Two hernia recurrences (1.0%) were established, one in each group.

**Conclusion:**

At 1 year after hernia surgery, the use of self-gripping and self-adhesive meshes lead to successful pain reduction and quality of life improvement.

Trial registration.

ClinicalTrials.com NCT03734224.

## Introduction

Groin hernia is a common disease, with more than 20 million patients worldwide undergoing hernia surgery each year [1]. The tension-free Lichtenstein procedure with the use of a flat non-absorbable mesh is the standard open repair technique in inguinal hernia repair [1]. Due to the use of mesh, hernia recurrence rate after open inguinal hernia repair is low, 1.5–3%, depending on the follow-up time [2–7]. Also, post-operative complications, such as bleeding, surgical site infection, or seroma, are rare [2, 8, 9].

The main symptom caused by inguinal hernia is pain or discomfort in the inguinal area, occasionally affecting daily activities [1, 2]. The goal for inguinal hernia surgery is to relieve such pain and improve quality of life. However, 10–12% of the patients suffer from clinically significant chronic pain after surgery [1, 6, 7], which is currently the issue of biggest interest concerning hernia treatment. Chronic pain is defined as post-operative inguinal pain lasting more than 3 months after surgery [1]. Even if the cause of post-operative chronic pain is unknown, several risk factors have been observed in earlier studies [5, 10], such as patient dependent factors (young age or female patients) and severe early post-operative pain or pre-operative pain. Factors in the surgical technique associated with less post-operative pain are laparoscopic surgery and the use of light-weighted mesh [5, 7]. On the other hand, mesh fixation does no seem to affect chronic pain rates [3, 11].

Originally, the mesh was fixed using non-absorbable sutures in the Lichtenstein procedure [12]. Today, a variety of modifications has been made to improve post-operative outcome and to simplify mesh fixation such as glue fixation, self-gripping, or self-adhesive mesh. The self-adhesive mesh (Adhesix™) is a compound of non-resorbable mesh and a self-adhering gel of polyvinylpyrrolidone (PVP) and polyethylene glycol (PEG) [13], whereas the self-gripping mesh (Progrip™) consists of non-resorbable mesh and resorbable micro grips [10]. Several trials and meta-analyses [3, 4, 6, 11] have evaluated sutured mesh with self-gripping mesh or glue fixation of the mesh and imply that glue fixation causes less pain in the early phase after the operation. Still, chronic pain seems to occur equally. Even though the self-adhesive mesh is commonly used and may be equalized with the use of glue fixation of a mesh, there is only a small number of studies examining it [13].

The aim of the present study was to evaluate post-operative pain after open inguinal hernia surgery using two different self-fixated meshes. The short-term results of our randomized trial [8] demonstrated an advantage of self-adhesive mesh over self-gripping mesh with respect to acute post-operative pain. Here, we present the results of the 1-year follow-up. To our knowledge, there are no prior randomized controlled trials comparing the postoperative outcomes of self-adhesive and self-gripping mesh.

## Material and methods

### Study design

This was a prospective, randomized clinical trial. Patients were randomized in parallel groups, using two different self-fixating meshes in open inguinal hernia repair. Two surgical units in Finland, Helsinki University Hospital and Päijät-Häme Central Hospital, participated in this study from November 2018 through to March 2021.

The study was approved by the ethics committee of Helsinki University Hospital (HUS/459/2018) and registered at ClinicalTrails.gov (NCT03734224). According to the study plan, an interim analysis was performed halfway through the study for safety reasons as well as to determine whether one of the groups had significantly better results than the other necessitating preterm stopping of the patient recruitment.

### Inclusion criteria

Male patients aged from 18 to 85 years, suitable for day case surgery and presenting a symptomatic primary unilateral inguinal hernia, were included in this study.

**Fig. 1:**
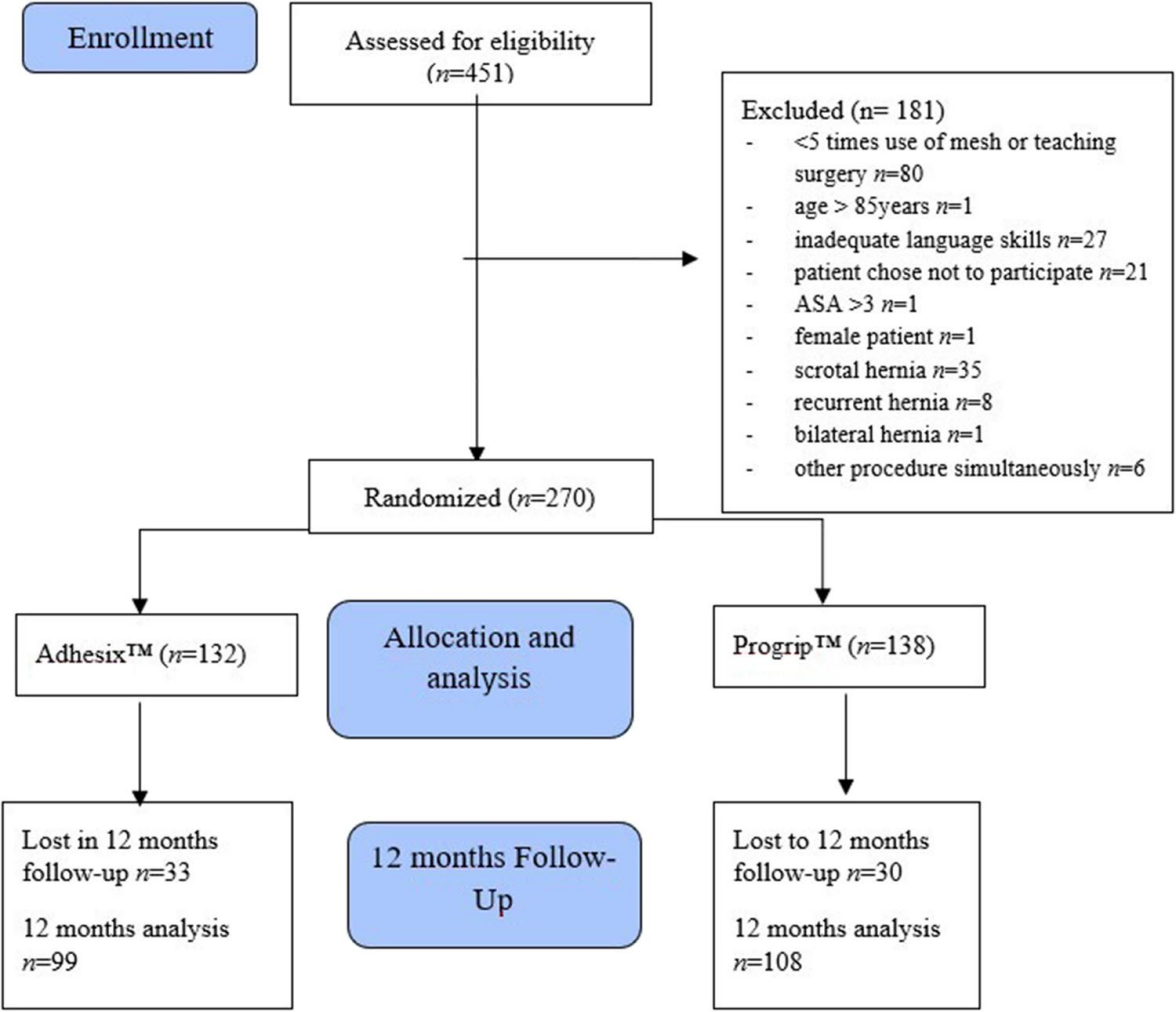
Flow diagram of the study design

Exclusion criteria were bilateral, recurrent, or incarcerated hernia, an American Society of Anaesthesiologists physical status (ASA) of IV or more, and a body mass index higher than 35 or lower than 18. Also, teaching surgery (a surgical trainee performs a portion of the entire operation under the guidance of a consultant) or cases, where the surgeon was inexperienced with both meshes (a minimum of five times use of both meshes was required) led to patient exclusion. Patients with inadequate language skills or patients not willing to participate were not included. The excluded patients were recorded (Fig. 1).

The participants received oral and written information about the study and signed an informed consent.

### Randomization

Each center performed block randomization in blocks of 10 participants. Numbered and sealed opaque envelopes were opened by the operating surgeon just before the procedure. Patients were enrolled consecutively and randomly received one of the treating options: the self-adhesive mesh (Mesh Adhesix™, 7.5 cm × 15.5 cm, Cousin Biotech, France) or self-gripping mesh (Progrip™, self-gripping polypropylene mesh, 12 cm × 8 cm, Covidien) in an otherwise equal operation.

### Operative technique

The operating surgeons (*n* = 8) were experienced in open hernia surgery. The surgery was done preferably under local anesthesia, alternatively under general anesthesia, if local anesthesia was insufficient or at patients request. The operating surgeon administered the local anesthetics (Ropivacaine). Routine operative technique was used, with an anterior approach to the inguinal area. Indirect hernia sacs were dissected, and resected, and direct hernias were inverted. The regional nerves were preserved when possible. No additional mesh fixation was used, and the self-fixated mesh was placed on the pubic bone, the inguinal ligament, and the internal oblique aponeurosis.

### Primary and secondary outcomes

The number of patients needing unplanned visits to a physician in the health care center (operating hospital or public and private health care centers) due to pain after the operation during the first three months was the primary outcome for this trial. Different measures of pre- and post-operative pain, i.e., the intensity of pain (measured by the numeric rating scale, NRS, ranging from 0 to 10) and the use of pain medication, were secondary endpoints. Also, quality of life measures (measured by RAND-36 Item Health Survey [14]) and postoperative complications were evaluated.

### Patient data

Demographic data and pre-operative pain intensity were registered before the operation. The form of anesthesia, the duration of the operation, the hernia type, and the type of mesh used and possible nerve resection were recorded. Clinical examination was carried out before the operation. The follow-up at 1, 3, and 12 months after the operation was performed by inquiries collected by the surgeons. Additional evaluation at the outpatient clinic was performed only in patients with pain or complications needing treatment. All patients’ medical records were reviewed post-operatively.

NRS pain scale (from 0 to 10) was assessed at rest, when coughing and during physical activity before surgery and at follow-up. The NRS scale is a simple and commonly used pain measurement tool, from 0 to 10, where 0 being “no pain” and 10 “the worst pain imaginable.” The RAND 36-Item Health Survey [14] is scored on a 0 to 100 range, where the lowest and highest possible scores are 0 and 100, respectively. Scores represent the percentage of total possible score achieved. Each item in the survey contains eight health concepts: physical functioning, role limitations due to physical health problems (during the last 4 weeks), role limitations due to personal or emotional problems (during the last 4 weeks), energy/fatigue, emotional well-being, social functioning, bodily pain, and general health perceptions.

### Statistical analysis

Sample size estimation for this study was based on our earlier published retrospective study [15], indicating that approximately 216 patients should be included in each group in order to detect clinically relevant difference between the outcomes (*α* 0.05, *β* 0.80). With an estimated dropout rate of 10%, we aimed for 480 patients in total. Halfway, an interim analysis was conducted. A significant difference between the groups in primary outcome was found, and the recruitment of the patients was terminated.

Variables were tested for normality of distributions with the Kolmogorov–Smirnov test. For numeric data, the independent samples *t* test or Mann–Whitney was used. Comparison of categorical data between the groups was performed by the chi-square test or Fisher’s exact test. Data is presented as mean (standard deviation) for numeric data and as frequency for categorical variables. A value of *P* < 0.05 was considered statistically significant.

All statistical analyses were performed using IBM SPSS statistics version 27 (IBM, Armonk, New York, USA).

## Results

The recruitment of patients was discontinued in June 2021 since a formal interim analysis for the primary endpoints was performed with significant differences in pain. Data of 270 patients were reviewed, and the results of the 3-month follow-up were reported previously [8]. In May 2022, 207 patients (response rate 76.2%) had completed a 12-month follow-up, the results of which are presented here. All patients’ medical records were reviewed 1 year after the operation.

### Patient characteristics

Two hundred seventy patients were randomly assigned into two groups between November 2018 and March 2021, 132 of whom received Adhesix™ mesh (A group), and 138 Progrip™ mesh (P group).

Baseline characteristics and operating details were similar between the two groups, as shown in Table 1 and closer presented in our previous report [8].

**Table 1:** Baseline characteristics and operative details, *P* value (chi-square°, Student’s *T*-test*)

	Adhesix™ (*n* = 132)	Progrip™ (*n* = 138)	Total (*n* = 270)	*P* value
Age (years), mean (SD)	54.5 (15.2)	55.4 (14.7)	54.9 (14.9)	0.607*
BMI (kg/m^2^), mean (SD)	24.5 (2.6)	25.2 (3.2)	24.9 (3.0)	0.053*
ASA classification, *n* (%)				
I	79 (60.3%)	76 (55.5%)	155 (57.8%)	0.700°
II	44 (33.6%)	53 (38.7%)	97 (36.2%)	
III	8 (6.1%)	8 (5.8%)	16 (6.0%)	
Anesthesia, *n* (%)				
Local	109 (83.2)	95 (68.8)	204 (75.8)	**0.007°**
General	22 (16.8)	43 (31.2)	65 (24.2)	
Hernia type, *n*				
Direct (M1/M2/M3)	52 (5/41/6)	46 (6/37/3)	98	0.475°
Indirect (L1/L2/L3)	63 (33/29/1)	81 (42/39/0)	144	
Combined	16	11	27	
Nerve resection, *n* (%)				
Ileohypogastric	11 (8.3)	7 (5.1)	18 (6.7)	0.276°
Ilioinguinal	2 (1.5)	1 (0.7)	3 (1.1)	
Genitofemoral	0 (0)	2 (1.4)	2 (0.7)	
None	119 (90.2)	128 (92.8)	247 (91.5)	
Operation time (min), mean (SD)	41.7 (11.9)	41.7 (12.6)		0.936*

Values in bold indicate statistically significant differences

### Primary outcome

The primary outcome of this study was the number of patients needing follow-up visits in the health care center after the operation, due to post-operative pain in the inguinal area. During the first 3 months, the number of follow-up visits in the P group was significantly higher than in the A group (*n* = 19 vs. *n* = 4, *P* = 0.001), as presented in detail in our previous report [8]. At 1-year follow-up, additionally, 6 patients (5.6%) with the Progrip™ mesh and 3 patients (3.0%) with the Adhesix™ mesh suffered from pain leading to additional follow-up visits (*P* = 0.502). To assess the total number of patients needing additional follow-up during the first year after surgery, all patients’ (*n* = 270) medical records were reviewed. Twenty-two (15.9%) patients in the P group and 6 (4.5%) in the A group (*P* = 0.002) suffered from pain leading to an evaluation at the operating hospital or health care center, with the vast majority of visits taking place during the first postoperative month. Table 2 shows the total number of pain related follow-up visits.

**Table 2:** Follow-up visits. Number of patients needing additional follow-up visits due to pain after surgery. *p* Value: (^2^=chi square, º=Fischer)

Follow-up visit due to pain			*P* value
	Adhesix™ (*n* = 101)	Progrip™ (*n* = 101)	
1–3 months after surgery (*n*/%)	4/2.3%	19/14.7%	**0.001**^**2**^
	Adhesix™ (*n* = 99)	Progrip™ (*n* = 108)	
3–12 months after surgery (*n*/%)	3/3.0%	6/5.6%	0.502º
	Adhesix™ (*n* = 132)	Progrip™ (*n* = 138)	
All patients’ post-operative visits (*n*/%)	6/4.5%	22/15.9%	**0.002**^**2**^

Values in bold indicate statistically significant differences

### Secondary outcomes

**Fig. 2:**
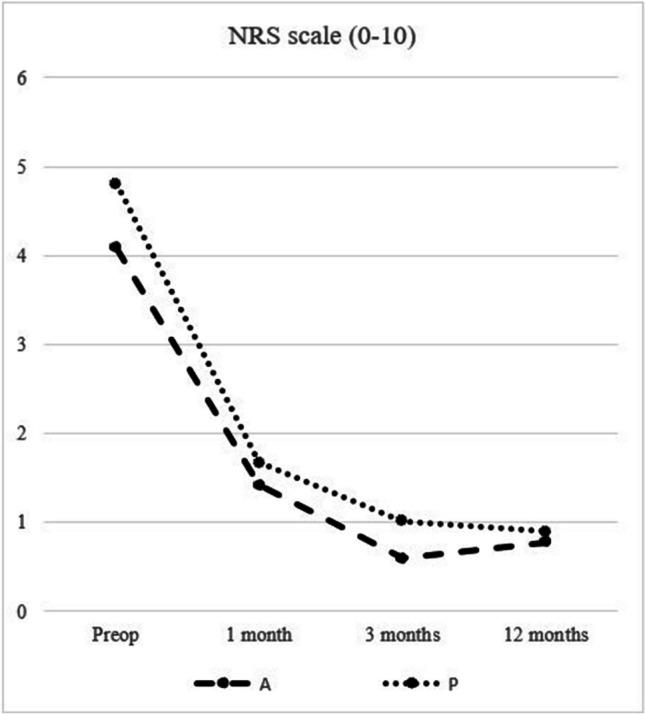
NRS scores (mean) during exercise preoperatively and at follow-up, A, Adhesix™; P, Progrip™

The degree of pain was estimated with the numeric rating scale (NRS) assessed at rest, when coughing and during physical activity. The mean NRS score at rest was low pre-operatively and at post-operative follow-up (A group 0.79–0.21, P group 0.97–0.34). At exercise, the mean NRS score was significantly improved at 1- and 3-month follow-up compared to the pre-operative state, after which it remained quite stable (Fig. 2). The mean difference in the NRS scores between the groups in favor for the A group at 3-month follow-up (A 0.60, P 1.03, *P* = 0.057) was diminished at 1-year follow-up (A 0.78, P 0.90, *P* = 0.410).

Patients with NRS 0 have no pain, whereas NRS 1–3 describe mild pain, NRS 4–6 intermediate pain, and NRS 7–10 severe pain. At 1-year follow-up, most patients (87.3%) had no pain at rest, additionally 10.7% of all patients suffered from mild pain (NRS 1–3), with no significant difference between the groups. In the A group, none of the patients had moderate (NRS 4–6) or severe pain (NRS 7–10) at rest, and in the P group, 4 patients (3.7%) reported moderate pain (*P* = 0.147). Further, during exercise, 5 patients (5.2%) with Adhesix™ and 8 patients (7.4%) with Progrip™ suffered from moderate or severe pain (*P* = 0.333).

The frequency of pain in the inguinal region was determined by a three-step scale; never, rarely, or frequently. Only 2 patients (1.0%) reported frequent pain at 1-year follow-up, while 80 patients (40%) rarely suffered from pain in the inguinal region. There was no significant difference between groups.

Similarly, the questions regarding pain in the RAND-36-Item Health Survey showed significantly less pain up until 3 months post-operatively (A 88.7, P 82.6) compared to the preoperative (A 70.8, P 63.0) and early post-operative state (A 72.5, P 63.7). After that time point, the severity of pain was stable without differences between the groups (A 87.1, P 82.5).

**Fig. 3:**
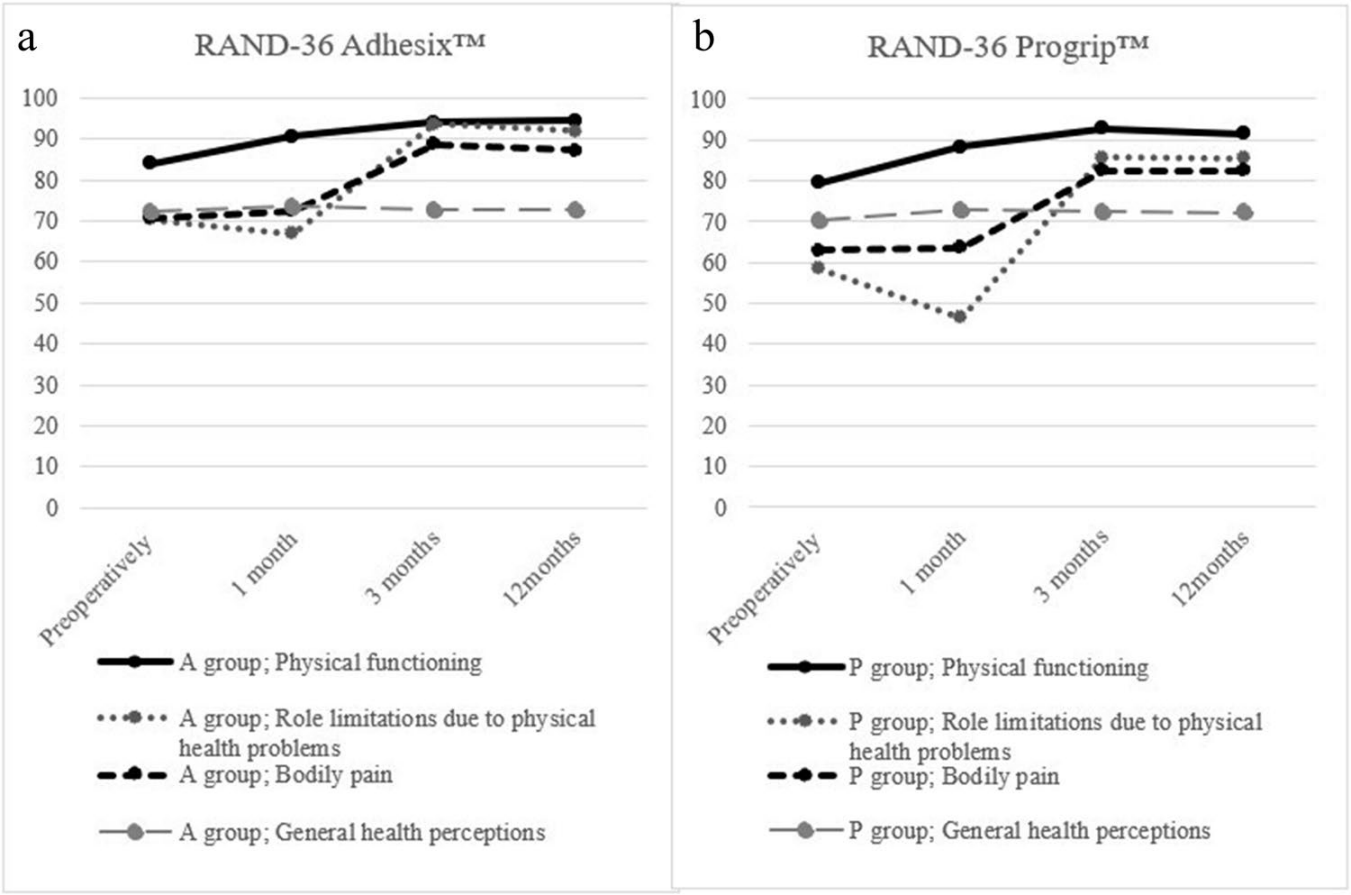
RAND 36-Item Health Survey before and after surgery (mean values, scored on a 0–100 range). **a** A-group = Adhesix™, **b** P-group = Progrip™

The dimensions in RAND-36 affected by pain are physical functioning and role limitations due to physical problems. At 1-year follow-up, both groups have high ratings in physical functioning (A 94.4, P 91.6) and only minor limitations due to physical problems (A 91.8, P 85.5). The RAND-36 survey results during follow-up are presented in Fig. 3.

Two hernia recurrences (*n* = 2, 1.0%), one in each group, were established at 1-year follow-up, one of whom had already been re-operated at 1-year follow-up. No other post-operative complications were detected. All early complications until 3 months after surgery were presented in our previous report [8].

## Discussion

A number of previous studies regarding postoperative pain after hernia surgery have focused on the impact of different meshes and fixation techniques on the acute and chronic pain. However, to our knowledge, this is the first study comparing the medium-term effect of self-gripping and self-adhesive mesh on the chronic pain after hernia surgery. Importantly, although short-term outcome [8] favored self-adhesive mesh in terms of postoperative pain and number of postoperative health care contacts due to postoperative pain, the level of pain at 1 year was comparable between meshes. Further, after 3-month convalescence, there was no difference in additional visits to healthcare units. The study was discontinued at the interim analysis halfways due to the differences in acute post-operative pain [8], and the smaller sample size may have some influence on the 1-year results presented here.

In general, patients have moderate pain due to inguinal hernia before surgery. This study present mean preoperative NRS scores between 4 and 5, equivalent to results in other trials [3, 11]. The traditional way to compare differences of pain between experimental groups is to compare the mean NRS values at a defined time point. At 1-year follow-up, mean pain scores are usually low, even less than one, as shown in a recent prospective study [3]. Also, our results are in line with these findings.

Further, we wanted to widen the perspective of postoperative pain to clinically important issues such as pain-related healthcare visits and category-based evaluation of pain recommended to be used by guidelines. The reality is, that even though mean pain scores in studies are low, a small number of patients suffer from considerable post-operative inguinal pain. The literature propose the use of pain scales such as numeric rating scale (NRS) or visual analogue scale (VAS) to assess pain intensity. Hjermstad et al. [16] showed that NRS and VAS scores are compatible. Chronic pain is defined as at least moderate pain affecting normal daily activities lasting more than 3 months after surgery [1], which correspond to pain scores higher than 3. The incidence of clinically significant chronic pain is reported to be 10–12%, decreasing with time [1]. In this study, at 1-year follow-up to 5–7.5% of the patients reported chronic pain with the use of self-fixated meshes, which are equal or even slightly less than earlier studies. Matikainen et al. [3] presented a 6–10% risk for VAS > 3 at 1 year by using suture fixation, glue fixation, or self-gripping mesh in open inguinal hernia surgery. Likewise, Eklund et al. [17] observed intermediate or severe pain at 1 year after surgery in 7.1% of the patients, and in a questionnaire study [18] with 1 652 patients from the Danish database, 8% of the study population suffered from moderate or severe pain during physical activity. Fortunately, as well as in our trial, the risk of severe pain seems to be low in many trials, 0.8–2.0% [1, 18].

Moreover, the advantage of self-adhesive mesh over self-gripping mesh, with respect to acute postoperative pain and faster recovery after surgery, which was detected in this trial [8], was no longer distinguished at 1 year after surgery when evaluating the degree of chronic pain. Glue fixation seems to cause less acute postoperative pain according to earlier studies [4, 5, 13]. The microgrips on the surface of the self-gripping mesh resorbs in approximately 12 weeks [9], and the irritation caused by them may partly explain the difference in short-term pain between these self-fixated meshes. Also, a Cochrane database systematic review [19] support these findings and indicates that glue fixation may reduce postoperative pain. Although, with a longer follow-up, there seems not be considerable differences between the self-fixated meshes. Another possible mechanism for the early difference in pain might be explained by the different biomechanical influences of the meshes [20]. The area of Progrip™ mesh is smaller than the area of Adhesix™ mesh. This may lead to greater migration of the mesh during the early recovery phase after operation and by this mechanism cause excess pain. On the other hand, higher gripping power of Progrip™ might compensate the effect caused by the smaller surface area.

The indication for operative treatment of inguinal hernias is mainly pain or discomfort in the inguinal area [1] and the aim for the surgery is to eliminate these and furthermore increase the quality of life. The RAND 36-Item Health Survey is a widely used questionnaire describing quality of life by creating a multidimensional profile of eight different health concepts [14]. As expected, the quality of life in this study was significantly improved at 1 year compared to the preoperative state when evaluating physical functioning and pain, including less limitations due to physical functioning, while other sub-areas were not altered. Supporting this, Nienhuijs et al. [21] proposed that almost one-third of the patients have limitations in daily leisure activities as a consequence of chronic pain. Wennergren et al. [22] established better quality of life scores at 1 year also after laparoscopic hernia repair, with resembling values to our result. This implies that patients benefit from surgery independent of the type of mesh or surgical technique, and we claim that both self-gripping meshes have a good outcome.

Except from pain-related issues, this study observed low recurrence rates; however, with an insufficient follow-up time, as only few recurrences are detected during the first year. Other research [2, 4, 10] agree, with a recurrence rate of 1.5–3%. Other clinically relevant late (> 3 months) complications were not seen in this project.

This study has some limitations. First, reliable pain measurement is difficult. Many factors influence the sensation of pain. Therefore, we tried to present clinically relevant measurement which directly affect the patient or the health care system. Second, not all patients responded to the follow-up, nor where they routinely checked-up after surgery, although, as we have a national electronic health care database, considerable post-operative problems would have been detected during the reviewing of patients’ medical records at one year after surgery. Third, due to ethical reason, the recruitment of patients was terminated half-ways affecting the total number of recruited patients. The smaller sample size might have led to the lack of statistical differences between the groups. Also, the use of block randomization may cause imbalance in terms of secondary diseases such as diabetes mellitus. The strength of this study is the randomized clinical design and the comparable follow-up between the groups. This data provide knowledge on postoperative pain and its influence on actual daily life during the first year after surgery.

## Conclusion

To conclude, the data from this study demonstrate that surgical treatment of inguinal hernia significantly reduce pain in the inguinal area and improves quality of life. Although the self-adhesive mesh was slightly preferable than the self-gripping mesh in the initial recovery, the meshes were equivalent at 1-year follow-up, and the use of either mesh is appropriate in open inguinal hernia repair.
